# Complex Aortic Interventions Can Be Safely Introduced to the Clinical Practice by Physicians Skilled in Basic Endovascular Techniques

**DOI:** 10.3390/life12060902

**Published:** 2022-06-16

**Authors:** Sarolta Borzsák, András Szentiványi, András Süvegh, Daniele Mariastefano Fontanini, Milán Vecsey-Nagy, Péter Banga, Zoltán Szeberin, Péter Sótonyi, Csaba Csobay-Novák

**Affiliations:** 1 Department of Interventional Radiology, Semmelweis University, 1122 Budapest, Hungary; borzsak.sarolta@med.semmelweis-univ.hu (S.B.); sz.andris1@gmail.com (A.S.); suviandris@gmail.com (A.S.); fontanini.daniele@med.semmelweis-univ.hu (D.M.F.); vecsey_nagy.milan@med.semmelweis-univ.hu (M.V.-N.); 2 Semmelweis Aortic Center, Heart and Vascular Center, Semmelweis University, 1122 Budapest, Hungary; banga.peter@med.semmelweis-univ.hu (P.B.); szeberin.zoltan@med.semmelweis-univ.hu (Z.S.); sotonyi.peter@med.semmelweis-univ.hu (P.S.); 3 Department of Vascular and Endovascular Surgery, Semmelweis University, 1122 Budapest, Hungary

**Keywords:** aortic aneurysm, endovascular aneurysm repair, stentgraft, fenestrated, branched

## Abstract

Our purpose was to evaluate the risk associated with the learning curve of starting a complex aortic programme in an Eastern European country. A retrospective study was conducted involving the initial 20 patients (16 males, mean age: 65 ± 11 years) undergoing fenestrated/branched endovascular aortic repair in a single centre. Demographic, anatomical, procedural, and postoperative variables were collected. Our elective patient cohort consisted of 9 pararenal aneurysms (45%) and 11 thoracoabdominal aortic aneurysms (55%), with the latter including 4 chronic dissection cases (20%). A total of 71 branch vessels were incorporated (3.5 ± 0.9 per patient). The per vessel technical success rate was 100%. In-hospital mortality was 5% (1/20). At an average follow-up of 14 ± 22 months, the primary clinical success rate was 45% (9/20) and the secondary clinical success was achieved in 75% of cases (15/20). All-cause mortality at 14 months was 20% (4/20; aortic related: 1/20, 5%). Four bridging stent occlusions were found (5.6%). Mortality and reintervention rates were comparable to the initial results of high-volume centres, while the complexity of our cases and the per vessel technical success rate was comparable to the values reported as late experience. The morbidity of the learning curve could be decreased if operators are skilled in basic endovascular procedures.

## 1. Introduction

Technical developments of endovascular aortic repair (EVAR) and visceral stenting nowadays allow the safe and durable treatment of the visceral aortic segment. Since the first implantation of a fenestrated stent graft in 1998 by Anderson, significant advantages of fenestrated/branched endovascular aortic repair (FBEVAR) have been shown regarding mortality and morbidity compared to open surgical repair [1,2,3]. FBEVAR has been widely adopted in several countries during the last decade, which provided scientific evidence to support the recent guideline recommendations of the European Society for Vascular Surgery favouring FBEVAR over open surgery for patients with a suitable anatomy [4]. However, there are significant regional and geographical alterations regarding the availability of such therapies, especially in Eastern Europe [5,6]. These disparities can either be attributed to the lack of experience of the centres in association with incomplete or absent centralization, or due to reimbursement and/or availability issues of such devices. Nonetheless, Eastern European endovascular practice is largely missing in the current literature. The aim of our study was to evaluate the risk associated with the learning curve of starting a complex aortic programme in a pioneer centre of Hungary.

## 2. Materials and Methods

This study is a single centre retrospective analysis conducted under the Semmelweis University Regional and Institutional Committee of Science and Research Ethics 96/2021. Current analysis includes our first 20 consecutive patients treated with FBEVAR. Informed consent was obtained from each patient.

### 2.1. Data Collection

Cardiovascular risk factors, demographics, anatomical, procedural, and postoperative variables were collected retrospectively. Follow-up clinical examination and imaging for all patients included in the complex endovascular aortic programme was performed at baseline, 30 days, 3 to 6 months, 12 months, and annually thereafter. Imaging included computed tomography angiography (CTA), magnetic resonance angiography (MRA), duplex ultrasound (DUS), or contrast-enhanced ultrasound (CEUS) studies. Intraoperative cone-beam computed tomography (CBCT) was used to confirm technical success whenever possible.

### 2.2. Data Analysis

Terminology, measurement techniques, and endpoint definitions were used according to the reporting standards of the Society for Vascular Surgery. This recently published document defines technical success if arterial access, delivery, and deployment of the stent graft, side branch cannulation, and the placement of the bridging stents are all successful, and if all target vessels are patent and there is no sign of type I or III endoleak on the 30-day follow-up CTA [7]. Primary endpoints were in-hospital and late aortic mortality and major adverse events, including the composite endpoints of all-cause mortality, new-onset dialysis, paraplegia, bowel ischemia, myocardial infarction, major stroke, or respiratory failure. Any unanticipated procedure performed after the index procedure was considered a secondary intervention, further classified as major if open surgery or large-bore (≥12 Fr) endovascular access was needed, and minor if percutaneous ≤10 Fr access was obtained. Categorical variables were reported as total numbers and percentages and continuous variables as means with standard deviations. Time-dependent variables were reported using the Kaplan–Meier method. Statistical analyses were carried out using IBM Corp. Released 2020. IBM SPSS Statistics for Windows, Version 27.0 (IBM Corp., Armonk, NY, USA) and GraphPad Prism 8 (GraphPad Software, San Diego, CA, USA), and the latter was also used to graph data.

### 2.3. Technique

After the initial three cases, all procedures were performed by the same interventional radiologist (CCN). The primary operator is a proctor for the majority of aortic device companies and has 13 years of experience in endovascular procedures. The aortic team consists of another interventional radiologist (7 years of experience) and two diagnostic radiologists (4 and 6 years), two vascular surgeons with >20 years of experience in thoracoabdominal open repair, two cardiac surgeons with 35 and 15 years of experience in aortic surgery, and a cardiovascular anaesthesiologist with 10 years of experience. All of our team members are working in an institute dedicated to cardiovascular care. All procedures were performed under general anaesthesia in a hybrid endovascular room with a fixed imaging system. Decision on stent design was based on centreline analyses performed by the primary operator (CCN) using 3Mensio Vascular software (Pie Medical Imaging B.V., Maastricht, The Netherlands). Off-the-shelf (OTS) branched stent graft (Cook t-Branch, Cook Medical Inc., Bjaeverskov, Denmark) or patient-specific custom-made devices (CMDs) with up to five fenestrations or branches (Cook Medical Inc., Bjaeverskov, Denmark and Terumo Aortic, Inchinnan, UK) were used in the following fashion: reinforced fenestrations were preferred for vessels originating from narrow aortic segments, whereas directional branches were used to incorporate vessels that originate from wide aortic segments. Fenestrations were aligned to target vessels with balloon-expandable covered stents (Begraft Peripheral or Begraft Peripheral Plus, Bentley InnoMed GmbH, Hechingen, Germany; Atrium V12, Getinge AB, Gothenburg, Sweden; Viabahn VBX, W.L. Gore & Associates, Newark, DE, USA), whereas directional branches were bridged to target vessels with balloon-expandable (Begraft Peripheral or Begraft Peripheral Plus, Bentley InnoMed GmbH, Germany; Viabahn VBX, W.L. Gore & Associates, USA) or self-expandable covered stents (Viabahn, W.L. Gore & Associates, USA; Fluency or Covera, BD, USA), the latter being used in our early experience only. Stent selection was at the discretion of the interventionist but had been heavily influenced by device availability of the different devices and budget constraints at the time of implantation.

Open surgical cutdown was performed in all cases, suture-mediated closure devices not being reimbursed. A shift from transaxillary to transfemoral access can be observed through the years, with the latter being performed lately almost exclusively with the help of a 16F steerable sheath (Heli-FX Guide 22, Medtronic plc, Dublin, Ireland). Wire-loops to increase the support of the steerable sheath were not needed nor used.

Prophylactic cerebrospinal fluid drainage (CSFD) was used selectively in our early experience when the risk of paraplegia was deemed high, associated with either the extent of the repair or other parameters (subclavian artery patency, internal iliac artery patency, large number and/or large diameter of intercostals/lumbars to be occluded during the operation), based on aortic team decision. Lately, therapeutic CSFD is preferred due to the relatively high risk of adverse events associated with CSFD.

On-table extubation is preferred lately to check for neurological complications as early as possible. Postoperative period was primarily managed in a dedicated cardiovascular intensive care unit by intensivists and nurses experienced in the treatment of vascular disease and with a close collaboration with the primary operators. Lately, we prefer to manage the postoperative period in the vascular surgery department with close supervision and with the help of the intensivists only if needed. This helped to reduce complications associated with the ICU stay.

## 3. Results

Among the initial 20 cases (16 men, 65 ± 11 years) enrolled in this study and treated by FBEVAR, there were 9 pararenal aneurysms (PRA, 45%) and 11 thoracoabdominal aortic aneurysms (TAAA, 55%), the latter including 4 chronic dissection cases (20%). There was no Marfan syndrome patient in this group, and all aneurysms were degenerative. Demographics, clinical, and anatomical characteristics are shown in Table 1.

Procedural details can be found in Table 2. Average aortic coverage length was 346.6 ± 132.8 mm. Custom-made devices were used in the majority of cases (14/20, 70%). Seventy-one renal and mesenteric vessels were incorporated with forty-six fenestrations and twenty-five directional branches. Two cases (10%) were managed via transaxillary access, after which we shifted to a transfemoral only approach using a 16 Fr steerable guide catheter to facilitate target artery cannulation (Heli-FX Guide 22, Medtronic plc, Ireland). All target arteries were successfully cannulated and stented and no open surgical conversion was needed. CSFD was used in three cases (15%) based on aortic team decision, two were prophylactic, one was therapeutic. The therapeutic-only approach was preferred lately over prophylactic insertion. Perfusion branches were used in four patients (20%) deemed high risk for spinal cord ischemia (SCI). Two out of the six OTS devices were used off-label, one due to a narrow visceral aortic segment and another with the chronic occlusion of the celiac trunk. The latter urgent case was managed with the occlusion of the corresponding portal using a combination of a covered stent and an Amplatzer plug (Amplatzer Vascular Plug II, Abbott Laboratories, Chicago, IL, USA) after antegrade and retrograde recanalization attempt of the celiac trunk through the gastroduodenal arcade both failed. Five cases were managed with six adjunctive procedures (5/20, 25%): two iliac bifurcation device implantations, two left subclavian transposition/bypass (zone 2 debranching), a prophylactic internal iliac artery recanalization, and a branch portal embolization. Preloaded catheters were not available and thus were not used.

**Figure 1:**
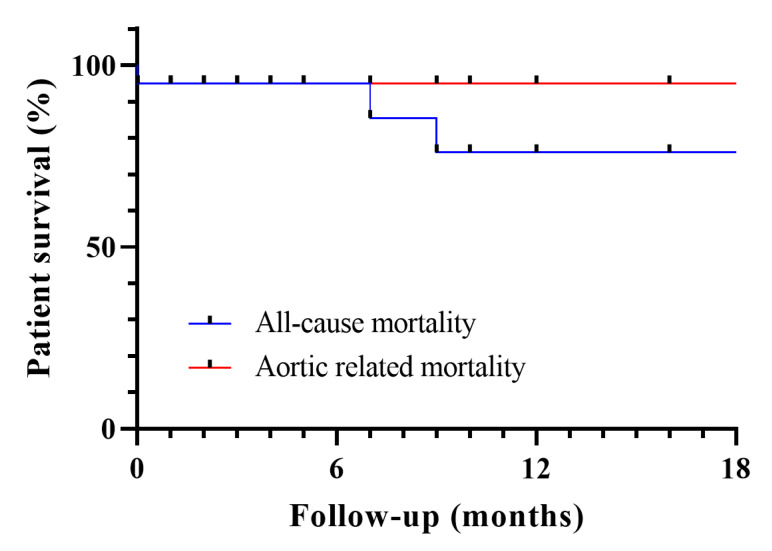

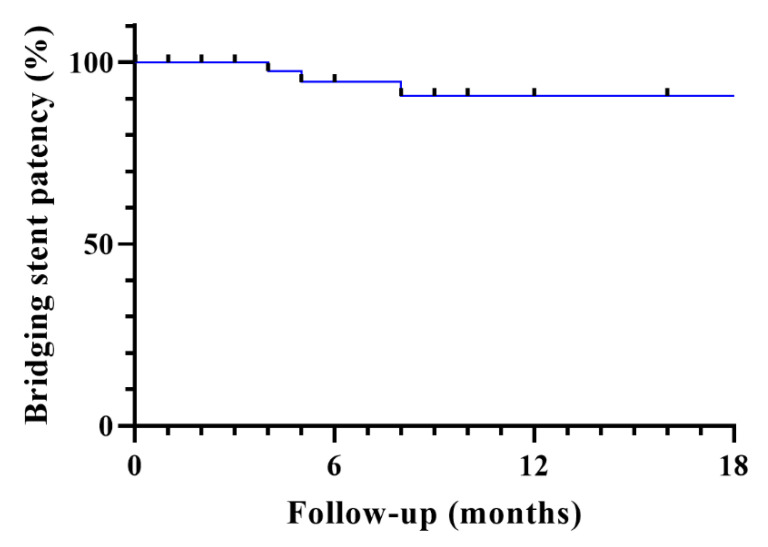
Patient survival (**upper**) and bridging stent patency (**lower**) at an average follow-up of 14 ± 22 months.

Average total length of stay (LoS) was 5.9 ± 2.4 days with an intensive care unit (ICU) LoS of 0.8 ± 1.2 days. Per patient technical success rate was 65% (13/20). Technical failure was mostly due to the need for an early reintervention (major: 1/20, 5%, minor: 5/20, 25%), with one in-hospital death associated with the unintended coverage of a common hepatic artery arising from the superior mesenteric artery. At an average follow-up of 14.0 ± 21.9 months, primary clinical success rate was 45% (9/20), whereas secondary clinical success was achieved in 75% (15/20). In-hospital mortality was 5% (1/20). All-cause mortality at 14 months was 20% (4/20), with only one case being aortic-related (5%). In that case, the coverage of an atypical common hepatic artery led to the patient’s death. One celiac and three renal stent occlusions were found (4/71, 5,6%, Figure 1).

Spinal cord injury occurred in two patients (10%), one paraplegia occurred in association with spinal epidural hematoma as a complication of a prophylactic CSFD, and a case of a paraparesis. Three cases of new-onset permanent dialysis were found (15%), two of them associated with renal stent occlusions. Aortic rupture, stroke, and myocardial infarction was not discovered.

## 4. Discussion

Initially developed to extend the indications of EVAR in high-risk patients with insufficient proximal landing zone, FBEVAR has recently matured to a widely accepted alternative to open surgery for complex aortic pathologies in all patients, regardless of surgical risk. Although FBEVAR is widely available for patients in developed countries, significant geographical disparities remain, especially in less-developed countries, e.g., the Eastern European countries.

The slower adoption of this technology can be attributed to several factors. A slow dissemination was seen in the US after the Food and Drug Administration approval of the Zenith Fenestrated endovascular graft (ZFEN, Cook Medical) in 2012, with 30% of the physicians who received ZFEN training not ordering a single device and 80% ordering <five devices/year [8,9]. The greater technical complexity of these demanding procedures requiring advanced endovascular skills, greater reliance on a complicated preoperative planning, and the need of advanced imaging equipment were identified as barriers of more widespread use, resulting in a reduced number of cases performed [8]. The need for a highly specialised imaging and for precise complex planning limiting the adaptation of FBEVAR techniques as the procedural planning requires not only measurements but extensive knowledge of the parameters, which affect device delivery, deployment, and target artery cannulation [10]. Furthermore, restricted access to appropriate devices remains a limiting factor in the adoption of novel endovascular techniques in the Eastern European region. Dissemination of the FBEVAR technique is even slower in the vast majority of Central and Eastern European countries, with a very few exceptions only. In Hungary, the missing reimbursement of complex aortic procedures is paired with the lack of centralisation due to political reasons, despite the provided evidence, resulting in less than 10 complex aortic procedures performed altogether by the tertiary centres until 2019 [11]. Being a tertiary vascular centre in Hungary, we established the first Aortic Centre of Hungary in early 2020, which resulted in an outbreak of complex aortic procedures compared to earlier years, despite the ongoing struggle with the limited budget for aortic procedures. Centralisation and treating patients in multidisciplinary teams provide a better care for the patients. Alberga et al. showed an association of hospital volume with perioperative mortality of complex endovascular repairs. In this Dutch nationwide study, they detected a perioperative mortality following FBEVAR in 9.1% in hospitals with a yearly volume of <9, while 2.5% in hospitals were performing more than 13 complex endovascular aneurysm repairs [12].

Despite having a case load (ca. 10/year) way less than ideal (3/month) to achieve lower adverse event rates, we were able to deliver an outcome at least noninferior to the most experienced centres of open repair of TAAA [13]. The in-hospital mortality rate found in our initial series (5%) compares favourably to the operative mortality of 6.2% reported by Coselli et al., a benchmark of elective thoracoabdominal open repair [14]. Mirza et al. reported the learning curve at the Mayo Clinic with a 6% mortality rate of the initial patient cohort (*n* = 81), while Schneider et al. reported a similar 6% perioperative mortality rate of the initial 50 FBEVAR cases performed in New York-Presbyterian Hospital. Our mortality rate compares well to these data, especially since our initial patient cohort, and thus our experience, is much smaller than that of the cited authors’ [8,13]. Furthermore, we achieved a lower in-hospital mortality rate than reported in the WINDOWS trial (10.1%), a study that was planned to minimize centre effect and evaluate the real-world mortality of FBEVAR [15]. However, some authors reported lower mortality rates, e.g., Schanzer et al. evaluated their single centre’s experience of the first 100 consecutive FBEVAR of complex aortic aneurysms. In their observational cohort study, a mortality rate of 3% was shown [10].

Staged repair, which was performed in one-fifth of our cases, is widely accepted to be associated with reduced rates of neurological complications such as paraparesis and paraplegia. These neurologic symptoms remain a dreaded complication of extensive aortic repair [16].

Initial risk associated with the starting of a complex aortic programme is heavily dependent on the operating team’s skills. Previous experience with EVAR and crural angioplasty may be associated with a steeper learning curve [17]. Our centre has more than two decades of experience in aortic interventions, with numbers approaching 100/year in the last five years. We were also one of the very first centres in Europe to perform angioplasty of the branches of the aorta, a skill that is essential to achieve success in complex aortic repair [18]. Up to now, no unconnected fenestrations/branches occurred, resulting in a 100% per vessel technical success rate, which is rather unusual in the initial cases of a newly established centre [19]. In comparison, Schanzer et al. reported a 2.3% failure to cannulate and bridge any targeted artery resulting in a 97.7% per vessel technical success rate [10]. Per patient technical success rate (65%) was compromised mostly by a rather high reintervention rate (30%), although the vast majority of these were classified as minor and values compare well with literature data [8,13].

It is well known that early experience usually involves very high-risk patients unfit for open repair [8,13,17]. Our initial cohort almost exclusively consisted of patients deemed unfit for major surgery, 95% of them being ASA class III-IV, which is among the highest values reported in association with early experience [13]. It is also usual that the complexity of the FBEVAR implants increases with the growing experience of a team [13,17]. The trends regarding the complexity of our repairs cannot be evaluated due to our very small patient cohort; however, more vessels were incorporated per patient (3.5 ± 0.9) in our present study than in the early period of Mirza et al. (2.8 ± 0.9) [13]. Prior aortic surgery (50%) and postdissectional TAAA repair (20%) occurred with a frequency that is comparable to that reported by Oderich et al. very recently, also suggesting that the complexity of our initial cases was somewhat higher than what is usual to start with [20].

There are limitations of our study. This is a single-centre, retrospective study which includes only twenty FBEVAR cases and has a relatively short follow-up. Since FBEVAR has several long-term complications, a longer follow-up would be necessary to evaluate the durability of these repairs. The lack of a standardized approach for patient selection might also result in patient and material selection. Furthermore, the heterogeneity of the patients regarding the gender and the pathology treated also limits the generalisability of our findings.

## 5. Conclusions

The initial outcome of our complex aortic programme showed high technical success and a low complication rate with a high freedom from disease-related mortality. Mortality and reintervention rates were comparable to the initial results of high-volume centres, while the complexity of our cases and the per vessel technical success rate are comparable to the values reported as late experience. The late addition of FBEVAR to our treatment portfolio and the advanced skills of our team in standard aortic and visceral interventions may have helped us to avoid the higher mortality and morbidity associated with the learning curve of our complex aortic programme.

## Figures and Tables

**Table 1:** Demographics, clinical, and anatomical characteristics.

Variable		*n* (%) or Mean ± SD
Demographics	Male gender	16 (80)
	Mean age, years	65.5 ± 11.2
	BMI, kg/m^2^	27.3 ± 4.1
Clinical Characteristics	Hypertension	16 (80)
	Smoking	8 (40)
	Hypercholesterolemia	10 (50)
	Diabetes mellitus	3 (15)
	Coronary heart disease	11 (55)
	Chronic obstructive pulmonary disease	7 (35)
	Chronic kidney disease stage III–V	4 (20)
	eGFR, mL/min/1.73 m^2^	74.6 ± 16.9
	Prior aortic repair	10 (50)
	Malignant disease	5 (25)
ASA status	ASA II	1 (5)
	ASA III	17 (85)
	ASA IV	2 (10)
Anatomical characteristics	Pararenal aortic aneurysm	9 (45)
	Thoracoabdominal aortic aneurysm	11 (55)
	Chronic dissection	4 (20)
	Average size of the aortic aneurysm, mm	72.5 ± 17.0

Abbreviations: *n* = Number; SD = Standard deviation; BMI = Body mass index; ASA = American Society of Anesthesiologist’s physical status classification.

**Table 2:** Procedural details.

Variable		*n* (%) or Mean ± SD
Device design	Off-the-shelf device	6 (30)
	Patient-specific device	14 (70)
Proximal sealing zone	zone 2–4	10 (50)
	zone 5	7 (35)
	zone 7	1 (5)
	zone 8	2 (10)
Distal sealing zone	zone 9	3 (15)
	zone 10	11 (55)
	zone 11	6 (30)
Aortic coverage length, mm		346.6 ± 132.8
Total incorporated vessels		71
Incorporated vessels per patient	Total	3.6 ± 0.9
	1 vessel	1 (5)
	2 vessels	1 (5)
	3 vessels	5 (25)
	4 vessels	12 (60)
	5 vessels	1 (5)
Type of incorporation	Fenestrations	46 (65)
	Directional branches	25 (35)
Procedural data	Contrast volume, ml	285.4 ± 124.0
	Fluoroscopy time, min	69 ± 39
	Cumulative air kerma, Gy	3.6 ± 2.5
ICU length of stay, d		0.8 ± 1.2
Total length of stay, d		5.9 ± 2.4
Staged repair		6 (30)
Cerebrospinal fluid drainage		3 (15)
Temporary aneurysm sac perfusion		4 (20)
Technical success per vessel		71 (100)
Primary technical success per patient		13 (65)

Abbreviations: *n* = Number; SD = Standard deviation; ICU = Intensive care unit.

## Data Availability

Not applicable.
